# Rapid clearance of inducible HIV-1 proviruses after initiation of antiretroviral therapy

**DOI:** 10.1371/journal.ppat.1013466

**Published:** 2025-09-11

**Authors:** Maria C. Puertas, Lucía Bailón, Víctor Urrea, Maria C. García-Guerrero, Yovaninna Alarcón-Soto, Angel Rivero, Beatriz Mothe, José Moltó, Javier Martinez-Picado, on behalf of the DUALITY study group

**Affiliations:** 1 IrsiCaixa, Badalona, Barcelona, Spain; 2 Institute for Health Science Research Germans Trias i Pujol (IGTP), Badalona, Spain; 3 CIBERINFEC, Instituto de Salud Carlos III, Madrid, Spain; 4 Department of Infectious Diseases, Hospital Universitari Germans Trias i Pujol, Badalona, Spain; 5 Fundació Lluita contra les Infeccions, Badalona, Spain; 6 Projecte dels NOMS-Hispanosida, BCN CheckPoint, Barcelona, Spain; 7 University of Vic-Central University of Catalonia (UVic-UCC), Vic, Spain; 8 Catalan Institution for Research and Advanced Studies (ICREA), Barcelona, Spain; Duke University, UNITED STATES OF AMERICA

## Abstract

Despite its efficacy, antiretroviral therapy (ART) is not curative, and HIV-1 rebound occurs whenever treatment is interrupted. The viral reservoir in latently infected cells is the source of the infection resurgence, making it crucial to understand when and how this reservoir is established so that it can be targeted more effectively. In this study, we evaluated the decay dynamics of proviral DNA in 40 ART-naive people initiating dolutegravir-based treatment and compared these dynamics to the decay kinetics of inducible proviruses, as measured using the VIP-SPOT assay. Intensive sampling during the first month, followed by regular sampling up to 48 weeks, enabled us to outline the biphasic decay dynamics of different fractions of the viral reservoir. Our results show that the first decay phase of inducible proviruses is significantly faster than that of total HIV-1 DNA (2.6 days *versus* 5.1 weeks), indicating that selective pressure on this specific fraction of proviruses is particularly effective during the first days after ART initiation. These findings suggest that therapeutic interventions aimed at impacting the viral reservoir by boosting the immune response targeting the inducible fraction should be implemented at the time of, or immediately before, treatment initiation.

## Author summary

Currently, HIV infection is a manageable condition that can be controlled with antiretroviral treatment. However, people with HIV are not completely cured, as some immune cells can persist for many years containing a ‘dormant’ virus, that is viral DNA that keeps silent. This so-called viral reservoir makes people with HIV need lifelong treatment, as otherwise the virus can reawaken from these cells and start spreading again.

In this study, we tracked infected cells in the blood of 40 people from the time of treatment initiation and for the following 12 months. We found that once treatment begins, the clearance of cells containing the viral DNA is relatively slow, with a median half-life of 5 weeks. However, most of these cells contain viruses that cannot reactivate.

Importantly, we discovered that the small fraction of cells containing inducible virus -the kind that could restart the infection- is preferentially and rapidly eliminated within the first week of treatment. These findings suggest that this small but important fraction of the viral reservoir, which is a key obstacle to a cure, is particularly vulnerable during the early days of therapy. This opens a window of opportunity for targeted interventions aimed at achieving an HIV cure.

## Introduction

HIV-1 is a major global health issue, with more than 40 million people living with the virus worldwide [1]. Despite the potency of current antiretroviral therapy (ART) in suppressing viral replication to levels that are undetectable by standard laboratory assays, infection remains a chronic condition, since treatment interruption typically leads to viral rebound within a few weeks [2]. This HIV-1 persistence is due to the presence of cells harboring proviral DNA without expressing viral genes. These cells, known as the latent HIV-1 reservoir, are primarily memory CD4^+^ T cells, which can survive for years in a resting state until an appropriate stimulus reactivates the cell and the provirus it contains [3]. Once reactivated, a latently infected cell may begin producing new virions, which, in the absence of ART, can reignite viremia. Therefore, understanding how the viral reservoir is established and maintained over the years is crucial if we are to develop a cure for HIV-1.

Current knowledge indicates that the establishment of a seminal HIV-1 reservoir begins within the first 1–2 weeks after acute infection [4,5] and that latently infected cells exhibit long-term survival thereafter. This is evidenced by the fact that early treatment initiation does not prevent viral rebound if ART is interrupted, even among adults treated in Fiebig stage I [6]. Moreover, in newborns, where a predominantly naive CD4^+^ T-cell repertoire suggests a lower capacity for a long-lived HIV-1 reservoir to become established, the case of the Mississippi Baby demonstrated that HIV-1 persistence can lead to viral recrudescence after ART withdrawal, despite treatment being initiated only 1 day after birth [7].

Beyond the initial seeding, the latent HIV-1 reservoir continues to expand if viral replication is not prevented. Consequently, numerous reports indicate that the viral reservoir size increases over time. Therefore, initiating ART during chronic infection results in higher levels of proviral DNA, both in adults and in children [8,9]. Reducing the viral reservoir size is considered a crucial step towards a cure for HIV-1 infection, as a smaller viral reservoir size has been demonstrated to be positively correlated with the possibility of achieving durable ART-free HIV-1 remission, which is defined as the ability of the host to control viral replication after ART withdrawal (post-treatment control) [10]. Indeed, natural cases of long-term HIV-1 remission after ART share several features, including early ART initiation and reduced HIV-1 reservoir size [11]. Likewise, total HIV-1 DNA has been shown to inversely correlate with time to rebound upon plain treatment interruption or after interventions aimed at enhancing viral control [12,13].In addition to its size, the composition of the reservoir is of paramount importance. Most latently infected cells carry defective viral genomes, and only a minor fraction contain intact proviruses (full-length and non-hypermutated) [14], which might reignite viremia upon ART interruption. Despite the cumulative expansion of the viral reservoir during untreated HIV-1 infection, recent evidence indicates that there is a critical window period immediately following ART initiation that significantly shapes the composition of the proviral reservoir [15]. During untreated infection, defective proviruses accumulate. However, a major proportion of the intact reservoir is established shortly after ART initiation, as the intact variants dated close to time of initiation predominate in the replication-competent reservoir. Indeed, the reservoir turnover is more active during untreated infection and directly correlates with viral load, whereas the median half-life of latently infected cells is notably longer after ART initiation [16,17]. This difference may be driven by higher levels of CD4^+^ T-cell activation during periods of viremia, leading to the reactivation and clearance of latently infected cells, while CD4^+^ T-cell expansion is favored upon ART initiation. Of note, improved cytotoxic T-cell responses driven by immune recovery after viral suppression may also play a critical role in reshaping the viral reservoir.

There has been longstanding interest in evaluating HIV-1 decay dynamics following ART initiation, as this provides critical insights into the nature of persistent reservoirs. Beginning with the pioneering studies by Perelson et al. [18,19], mathematical modelling of plasma viremia and infected CD4^+^ T cells in blood have been instrumental in predicting the longevity of latently infected cells [16,20–22]. Nonetheless, a deeper understanding of the dynamics of the latent reservoir and the factors modulating its composition is still needed. In the last years, the implementation of digital PCR has contributed to advance this knowledge. Several recent studies have evaluated the dynamics of the intact and defective viral reservoirs, revealing that the intact reservoir is preferentially cleared within the first months after ART initiation and during long-term ART, possibly owing to continuous immune pressure [23–26]. However, most intact viruses are unable to reactivate from latency, as the site of proviral integration and other epigenetic factors may restrict proviral expression [14]. Therefore, we require additional information regarding the dynamics of the inducible reservoir to better understand the factors regulating its establishment [27,28].

In this prospective study, we explored the precise decay dynamics of inducible proviruses during the first year following the initiation of first-line ART in 40 participants in the DUALITY study, in which people living with HIV (PWH) started first-line ART with dolutegravir (DTG)-based dual or triple regimens [29]. To do so, we used the VIP-SPOT assay, an ELISpot-like assay aimed at quantifying the frequency of latently infected cells that are able to reactivate and produce viral proteins [30]. Longitudinal characterization of inducible proviruses was performed in parallel with measurements of viremia, total and intact proviral DNA, and cell-associated HIV-1 RNA. Our findings demonstrate that the pool of cells harboring inducible HIV-1 proviruses is specifically and dramatically targeted within the first weeks after ART initiation, with median half-lives significantly shorter than those of the rest of the viral reservoir. These results highlight the unique nature of the inducible viral reservoir and provide insights into why most intact proviruses are found to be in deep latency.

## Results

### Characteristics of study participants and longitudinal sampling

In this study, we analyzed the dynamics of reservoir decay in 40 participants who initiated first-line ART with DTG-containing regimens upon HIV-1 infection diagnosis. Demographic and clinical characteristics are summarized in Table 1. All participants were male at birth, with most identifying as men who have sex with men (87.5%), and the median age was 32 years [IQR = 27–35]. At baseline, median plasma viral load (pVL) was 4.5 log_10_ HIV-1 copies/mL [IQR = 3.9-4.9], with 9 out of 40 participants having pVL levels higher than 100,000 HIV-1 RNA copies/mL. The median CD4^+^ T-cell count was 472 cells/mm^3^ [IQR = 355–579]. After treatment initiation with either DTG + 3TC or DTG + FTC/TAF (n = 20 each), intensive blood sampling was performed within the first month (baseline, week 1, week 2, and week 4). Subsequent samples were collected at months 3, 6, and 12.

**Table 1:** Demographic and clinical characteristics of the participants at study entry.

Characteristic	N = 40
Age, years	31.9 (27.3 - 35.4)
Male at birth, n (%)	40 (100%)
Ethnicity, n (%)	
Caucasian	25 (62.5%)
Hispanic-Latino	14 (35.0%)
Other	1 (2.5%)
Transmission group, n (%)	
Heterosexual	4 (10.0%)
MSM	35 (87.5%)
Unknown	1 (2.5%)
Time since diagnosis of HIV-1 (weeks)	2.1 (1.6 - 2.9)
Plasma viral load, log_10_ copies/mL	4.5 (3.9 - 4.9)
Absolute CD4^ +^ count, cells/mm^3^	472 (355 - 579)
% CD4^+^	24.1 (19.8 - 32.8)
Absolute CD8 count, cells/mm^3^	864 (589 - 1084)
% CD8^+^	47.5 (39.1 - 55.2)
CD4^ + ^/CD8^ +^ ratio	0.5 (0.4 - 0.8)

MSM: men who have sex with men.

Data are expressed as median (interquartile range) unless otherwise indicated.

### Suppression of plasma viremia after ART initiation

**Fig 1:**
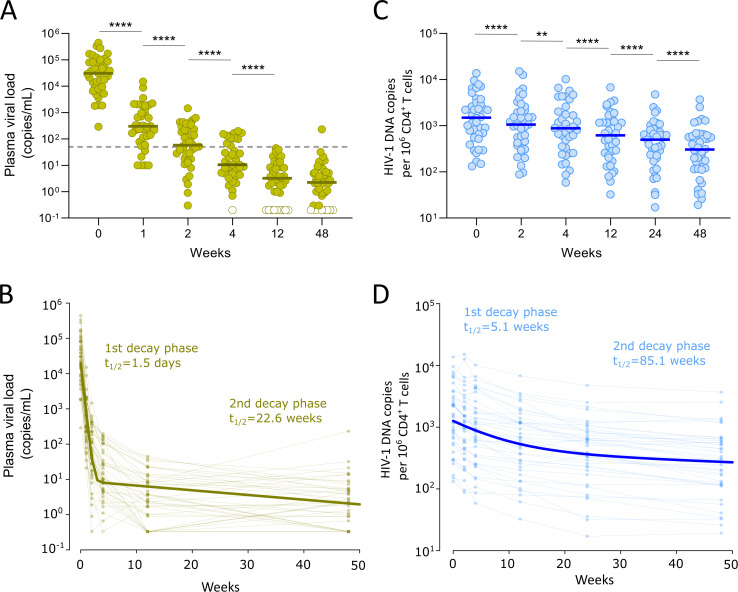
Decay of plasma viral load and proviral DNA. A. Hybrid plasma viral load (pVL) results: pVL at the initial time points was determined using standard assays (limit of detection: 20 HIV-1 RNA copies/mL). For each participant, once pVL became undetectable by this method, ultrasensitive quantification of pVL (limit of detection: 0.33 HIV-1 RNA copies/mL) was performed on all subsequent samples. Open symbols indicate undetectable pVL in the ultrasensitive assay. The dashed line indicates the 50-copies/mL level, and the median is shown for each time point. Asterisks indicate significant differences in pVL between consecutive timepoints (Wilcoxon matched-pairs signed rank one-tailed test: **** p-value<0.0001; *** p-value<0.001; ** p-value<0.01; * p-value<0.05). B. Biphasic decay dynamics of pVL fitted using non-linear mixed-effect models (dark green line). The half-life of pVL at each phase is indicated. Measured observations and connecting lines are shown in light green to illustrate interindividual variability. C. Absolute quantification of total HIV-1 DNA was performed using ddPCR in isolated CD4^+^ T cells for each participant and sampling time. The median is shown for each time point. Asterisks indicate significant differences in total HIV-1 DNA between consecutive timepoints (Wilcoxon matched-pairs signed rank one-tailed test: **** p-value<0.0001; *** p-value<0.001; ** p-value<0.01; * p-value<0.05). D. Biphasic decay dynamics of proviral DNA fitted using non-linear mixed-effect models. The half-life of infected cells at each phase is indicated. Individual longitudinal measures are shown in light blue to illustrate interindividual variability.

ART initiation led to rapid clearance of pVL in all participants, as expected with a second-generation integrase strand transfer inhibitor regimen. By the first week, the median viral load had decreased by 2 logs. By week 2, 45% of participants had pVL below 50 HIV-1 RNA copies/mL, and beyond week 12, all participants remained virally suppressed except for a single blip in one at the last timepoint (Fig 1A). To precisely delineate the dynamics of pVL below the limit of quantification with standard methods (20 HIV-1 RNA copies/mL in this case), plasma samples with undetectable data in those assays were re-tested using an ultrasensitive pVL assay with a limit of quantification of 0.33 copies per mL. Mathematical modelling of this hybrid pVL data set enabled us to investigate the longitudinal dynamics of residual viremia (Fig 1B). A first decay phase in pVL can be clearly delineated after ART initiation, with a half-life of 1.5 days. After a transition occurring beyond the first month, there is a second phase of decay in residual viremia, with a half-life of 22.6 weeks. Notably, only 5 participants (12.5%) had residual viremia levels ranging between 10 and 50 HIV-1 RNA copies/mL by the end of the study.

### Decay dynamics of cells harboring HIV-1 DNA after ART initiation

The same timepoints were evaluated to compare the decay dynamics of HIV-1–infected cells. Prior to treatment initiation, the proportion of CD4^+^ T cells containing HIV-1 DNA was 0.15% [IQR = 0.06%-0.31%]. As previously reported, these levels decayed slowly over the subsequent weeks (Fig 1C). Mathematical modelling of proviral decay indicates that a pool of short-lived infected cells leads the first phase, with a half-life of 5.1 weeks (Fig 1D). Subsequently, the survival of latently infected cells stabilized, with an estimated half-life of 85.1 weeks. At 48 weeks after ART initiation, the proportion of infected CD4^+^ T cells decreased by 81.6% compared to pre-treatment levels. Importantly, proviral levels at week 48 correlated significantly with those recorded at baseline (p < 0.0001, Spearman r = 0.88; S1A Fig).

**Fig 2:**
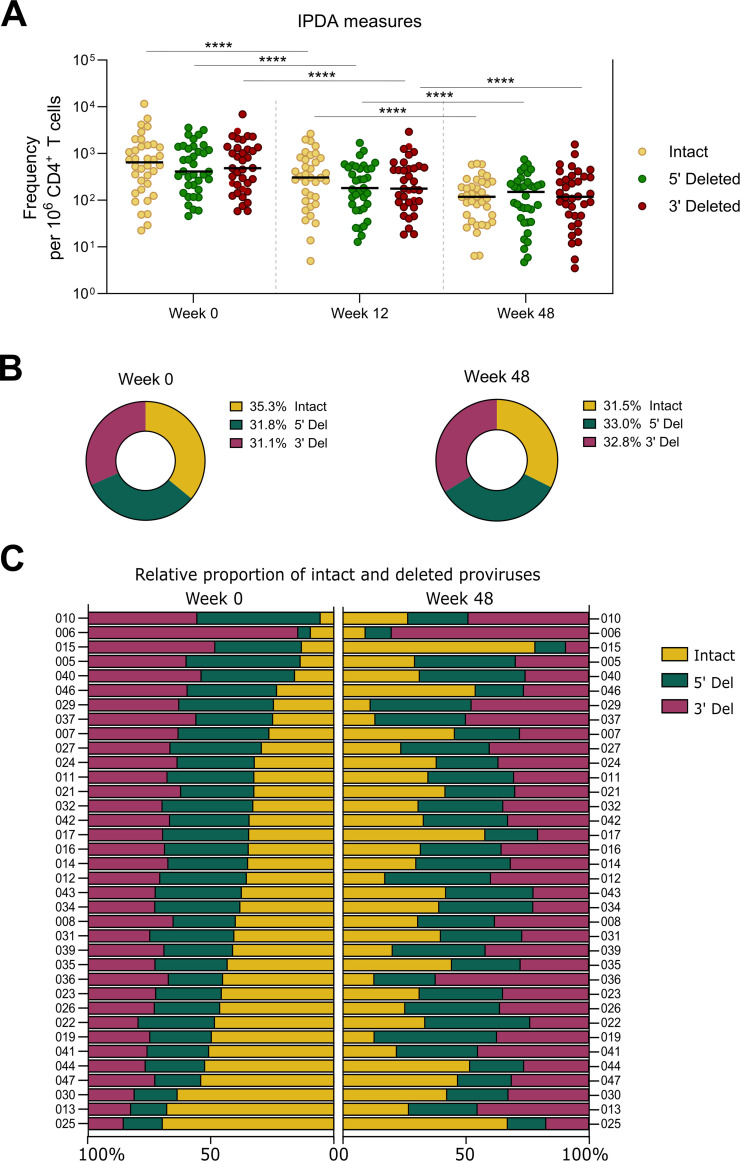
Evolution in the proportion of intact and defective HIV-1 proviruses in the first year after ART initiation. A. The absolute quantification of intact, 5’-deleted and 3’-deleted proviral genomes in peripheral CD4^+^ T cells was performed using IPDA. Asterisks indicate significant differences in the different HIV-1 DNA forms between consecutive timepoints (Wilcoxon matched-pairs signed rank one-tailed test: **** p-value<0.0001; *** p-value<0.001; ** p-value<0.01; * p-value<0.05). B and C. Relative proportion of intact, 5’-deleted and 3’-deleted viral genomes at weeks 0 and 48 is shown globally (median) and for each participant, respectively. In C, data from participants are ordered according to their relative proportion of intact proviruses at week 0.

As the predominant portion of the proviral reservoir consists of defective proviruses, which either contain large deletions or are hypermutated, we also evaluated the evolution of intact and defective proviruses following suppression of viral replication. According to the overall decay of HIV-1–infected cells, the frequency of cells harboring intact proviruses decreased by a median of 83.7% during the first year after treatment initiation (p < 0.0001, Fig 2A), and absolute levels of intact proviral DNA at week 48 correlated with those measured at baseline (p < 0.0001, Spearman r = 0.87; S1B Fig.). Regarding the relative proportion of intact proviruses, the median was 35.3% [IQR = 5.5%-69.7%] before ART initiation (Fig 2B). Proviruses with defects at the 5’ or 3’ ends were distributed proportionately in most cases. After 48 weeks of ART, the proportion of intact proviruses did not change significantly in the whole cohort, with a median of 31.5%. However, at the individual level, changes in this proportion were observed, either through contraction (in 64% of the participants) or expansion of intact proviruses, as shown in Fig 2C, thus illustrating divergent evolution patterns in the proportion of intact reservoir during the first year after ART initiation. Interestingly, we observed a significant association between lower baseline levels of HIV-1 DNA (both total or intact) and an increase in the proportion of intact proviruses after 48 weeks of treatment (S1C, D Fig).

### Evolution of viral transcription levels

**Fig 3:**
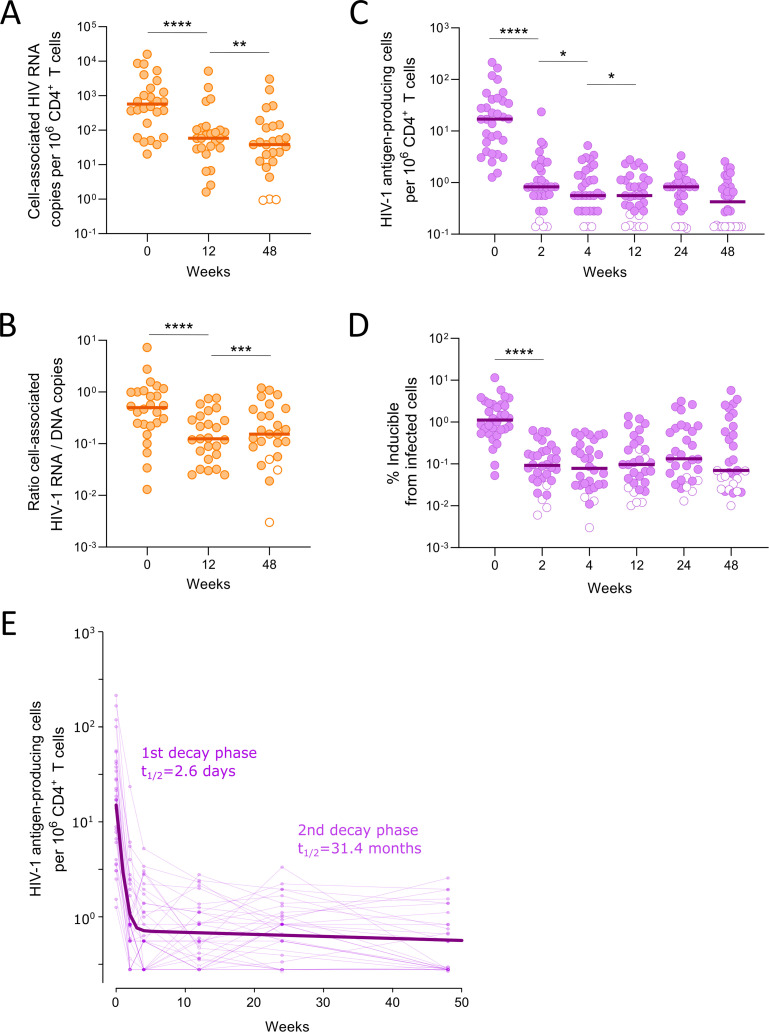
Changes in spontaneous viral transcription and the size of the inducible reservoir. A. The absolute quantification of cell-associated HIV-1 RNA copies is normalized by the total number of CD4^+^ T cells assayed. B. The transcriptional activity of infected cells is inferred by normalizing the total HIV-1 transcripts according to the frequency of infected cells in each sample (ratio of HIV-1 RNA to DNA copies). C. Quantification of HIV-1 antigen–producing cells upon *in vitro* activation is measured using the VIP-SPOT assay. D. The inducibility of infected cells is calculated by normalizing the number of reactivated cells according to the frequency of infected cells (those containing HIV-1 DNA) in each case. In figures A-D, median values are shown, and open symbols represent undetectable results, for which the value reported corresponds to half of the limit of detection calculated for each sample based on sample input. In A-D asterisks indicate significant differences between consecutive timepoints (Wilcoxon matched-pairs signed rank one-tailed test: **** p-value<0.0001; *** p-value<0.001; ** p-value<0.01; * p-value<0.05). E. Biphasic decay dynamics of the inducible reservoir fitted using non-linear mixed-effect models (dark purple line), with the half-life of the inducible reservoir at each phase indicated. Individual longitudinal measures are shown in light purple to illustrate interindividual variability.

To evaluate the dynamics of spontaneous HIV-1 transcriptional activity in infected cells, we measured HIV-1 RNA transcripts in peripheral CD4^+^ T cells throughout the study. A significant decay was observed, from a median of 575 cell-associated HIV-1 RNA copies per million CD4^+^ T cells [IQR = 135–1920] at baseline to a median of 38 copies [IQR = 12–137] at week 48 (Fig 3A). As this reduction is partly attributable to the decrease in the frequency of infected cells, we normalized these data based on the proportion of cells containing HIV-1 DNA in each sample. This enabled us to gain information regarding the transcriptional activity of the infected cells. By doing so, we were still able to detect a reduction in cell-associated HIV-1 RNA by week 12 (Fig 3B). However, we observed constant values thereafter, suggesting that viral transcription levels are reduced upon viral control, likely owing to the concomitant mitigation of immune activation.

### Decay dynamics of inducible proviruses

Mechanisms other than initiation of HIV-1 transcription play a key role in the establishment and maintenance of viral latency through blockade of viral RNA elongation and post-transcription steps. These mechanisms further contribute to the scarcity of intact proviruses able to effectively reverse from latency [31]. Therefore, we aimed to delineate the precise decay dynamics of the inducible reservoir by using the VIP-SPOT assay. This method precisely quantifies latently infected cells capable of reactivating upon stimulation by anti-CD3/CD28 to produce viral proteins, yet in samples collected before ART initiation (lack of detection of spontaneous p24 production was previously shown [30]). This measurement was performed on samples from baseline and weeks 2, 4, 12, 24, and 48 after ART initiation. In baseline samples, the median frequency of cells capable of responding and producing viral protein *in vitro* was 17.1 per million CD4^+^ T cells [IQR = 4.5-40.5], that is, 1.1% of infected cells (Fig 3C and 3D). These levels were rapidly and drastically reduced thereafter, with a median of only 0.8 HIV-1 antigen–producing cells per million CD4^+^ T cells detected by week 2 (equivalent to 0.093% of infected cells). Thus, the decay of inducible proviruses is much faster than that of cells containing total or intact HIV-1 DNA, demonstrating a median 93% decay within the first 2 weeks after ART initiation and an estimated half-life of 2.6 days (Fig 3E). A slight decrease was observed thereafter (t_1/2_ = 31.4 months), with the percentage of inducible infected cells at week 48 correlating with baseline values (p = 0.006, Spearman r = 0.49; S1E Fig).

### Differential clearance rates of infected cells depending on the characteristics of the integrated provirus

**Fig 4:**
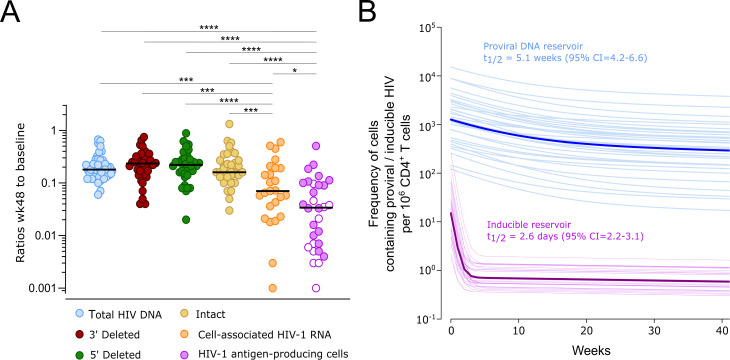
Differences in magnitude and velocity of decay of the different compartments of the viral reservoir. A. The overall decrease in each fraction of the viral reservoir during the first year after ART initiation is expressed as the ratio of week 48 to baseline values. Open symbols represent undetectable results at week 48. Significant differences in the magnitude of the decay of the different fractions of the viral reservoir are indicated with asterisks (Mann-Whitney test: **** p-value<0.0001; *** p-value<0.001; ** p-value<0.01; * p-value<0.05). B. Comparative first-phase decay dynamics of the total and inducible reservoir. Population decay trends fitted using non-linear mixed-effect models are shown in dark blue and purple lines for the total and inducible reservoir, respectively. Estimated half-life and 95% confidence intervals in the first-phase decay after ART initiation are indicated for each parameter. Individual-specific fitted decay dynamics are shown in light blue and purple lines to illustrate interindividual variability.

The data collected in this study enabled us to evaluate the differential magnitude and dynamics of decay in the pool of cells containing proviruses with various genetic and functional characteristics after treatment initiation. Over the first year of ART, both total and intact proviruses exhibited a similar overall decrease, namely, an 81.6% and 83.7% reduction, respectively, from the initial pool (Fig 4A). This contrasts remarkably with the massive depletion of 96.6% of cells containing inducible proviruses. Furthermore, mathematical modeling in our study clearly indicates that the initial decay phase of inducible proviruses is significantly faster than that of the total HIV-1 DNA reservoir (total proviral DNA t_1/2_ = 5.1 weeks, 95%CI = 4.2-6.6; inducible proviruses t_1/2_ = 2.6 days, 95%CI = 2.2-3.1 days; Fig 4B). Regarding intact DNA, our study determinations do not allow for such a precise definition of the decay rate after ART initiation. However, the 52% decrease observed 3 months after initiation of the study (Fig 2A) indicates that the decay slope is most probably similar to that observed for total DNA.

## Discussion

Comprehensive knowledge of the mechanisms modulating the establishment of the viral reservoir will provide insights into the nature of latently infected cells that survive in the long term. To this end, based on genetic and functional characteristics, we conducted a prospective follow-up of the different fractions of the viral reservoir to explore their relative abundance and define their differential decay dynamics in the first weeks after ART initiation and throughout the first year of treatment. In addition to PCR-based methods to quantify cells containing viral genomes—irrespective of whether they were intact or defective—we used the novel VIP-SPOT assay to precisely measure the frequency of cells retaining the ability to reactivate from latency and produce viral protein. Further insight into the establishment of the inducible reservoir may offer an additional perspective about the fate of cells that can be targeted by host immune responses.

First, compared to plasma viremia, we confirmed the slower decay of cells containing HIV-1 genomes in the first weeks after ART initiation. As previously noted, while pVL decreased by 2 logs within 1 week, we observed an initial decay phase in proviral DNA with a half-life of 5.1 weeks, followed by a subsequently slower decline [32,33]. The slower decay in infected cells than in plasma viremia suggests, as proposed by others [34], that a significant proportion of the virus in plasma originates from cells residing in lymphoid tissues rather than from the bloodstream, where our samples were collected. Next, we examined the genetic integrity of these viral genomes using the IPDA and found a decrease in the frequency of intact proviruses during the first year of ART that was very similar to the decrease observed for total HIV-1 DNA. However, because we did not analyze decay dynamics at very early time points after ART initiation, any potentially faster decrease in the initial weeks, as reported elsewhere, could not be captured here [25]. The proportion of intact proviruses was quite high at baseline (35%), as anticipated [35]. However, the finding of a stable fraction of intact proviruses after 1 year of suppressive ART in the whole cohort (32%) was quite unexpected. Of note, detailed analysis of the relative frequencies of intact and defective provirus in each participant revealed that, instead of a fixed proportion of intact viruses, the apparent overall stability of the intact reservoir resulted from divergent individual trends. Thus, while we observed a contraction of the intact fraction in some participants, others exhibited significant expansion after ART initiation. This finding could be attributed to homeostatic proliferation of CD4^+^ T cells following viral suppression or antigen-driven clonal expansion, which has been shown to be a driver of continuous alteration in the genetic repertoire of latently infected cells during ART [36,37]. Indeed, the association found between lower baseline levels of HIV-1 DNA and the increase in the proportion of intact proviruses may suggest that clonal expansion is a major contributor to the composition of the viral reservoir in participants with a smaller reservoir size. Although clonal expansion of cells carrying defective proviruses might be favored by the inability of the immune system to target them, it has been demonstrated that clones with intact proviruses may also become predominant and serve as a source of sustained low-level viremia, somehow evading clearance by host immune responses [38]. Despite this heterogeneity in the evolution of the intact reservoir in the first months after ART initiation, and drawing on observations from PWH under long-term viral suppression, we anticipate that the proportion of cells with intact provirus will preferentially decrease, possibly reflecting differential susceptibility to immune surveillance [23,24,26].

In addition to performing the genetic evaluation of viral reservoir dynamics, we investigated the fate of cells capable of reactivating upon stimuli and producing viral proteins, as this inducible subset of the viral reservoir may be influenced by immune selection mechanisms. The results presented here demonstrate that cells capable of reactivating from latency to produce viral antigens are preferentially and dramatically cleared within the first days following ART initiation, with a half-life of 2.6 days. This observation underscores the pivotal role of processes occurring during the first days after ART initiation in shaping the long-term viral reservoir. During viremia, continuous antigenic stimulation of immune cells promotes productive infections and rapid turnover of CD4^+^ T cells. In contrast, the transition to viral suppression boosts CD4^+^ T-cell expansion and provides a window of opportunity for cytotoxic T-cell responses to recover functionality. Within this period, CD4^+^ T cells can still be primed to produce viral antigens, making immunosurveillance pathways critical in selecting cells that persist among the pool of latently infected cells [39,40]. In this context, clones producing viral antigens, particularly immunodominant proteins such as Gag [41,42], are more susceptible to elimination by immune responses, irrespective of whether the provirus is fully intact or replication-incompetent. Contrariwise, proviruses with mutations that make them resistant to contemporary immune responses tend to persist. This may explain why rebound viruses and those captured during ART by using quantitative viral outgrowth assay (QVOA) are predominantly seeded during the window period preceding ART initiation and are prone to contain escape mutations [15,43–45]. Therefore, targeting these cells during their transition to the reservoir pool could be enhanced by therapeutic vaccines or broadly neutralizing antibodies that boost immune responses specifically during this critical “window of opportunity”. Such strategies could overcome current limitations and improve the targeting of proviruses that have evaded host immune responses [46].

This selection process occurring at ART initiation might also favor the survival of cells with proviruses integrated into regions that are non-permissive for transcription or those silenced by epigenetic factors [47,48]. This could explain why a significant fraction of the intact reservoir is not inducible *in vitro* and, therefore, not captured by QVOA [14]. Alternatively, the decay of the inducible reservoir may also result from a transition to deep latency driven by mechanisms yet to be characterized. Consequently, administering a latency-reversing agent capable of overcoming the blockade of viral transcription in these cells during this critical timeframe could be an effective strategy for limiting the establishment of the long-lived HIV-1 reservoir [40,49].

Longitudinal follow-up measurement of the frequency of replication-competent viruses after ART initiation in previous studies points to a biphasic decay of the inducible reservoir. However, the lack of frequent early sampling had precluded the characterization of this dramatic first-phase clearance [50]. Notably, further decay during long-term ART was not observed, either in this study or elsewhere [21], possibly owing to the selection of latently infected cells that are resistant to cell death pathways [51] or to proviruses integrated into host genes associated with cell growth [36,52,53].

The marked short half-life of inducible proviruses observed in our study may be also attributed to a high frequency of cells harboring unintegrated HIV-1 DNA (uDNA), existing in a state of pre-integration latency [54,55]. During untreated viremia, a significant proportion of infected CD4^+^ T cells can carry uDNA forms, either linear or circular. While direct expression of viral proteins from such extrachromosomal forms has been shown to be possible, it is generally inefficient. However, upon subsequent cellular activation, these forms may integrate into the host genome and initiate productive infection [56]. The stability of those unintegrated viral DNA molecules is controversial [57–62]. In our study, the VIP-SPOT assay may facilitate the transition from pre-integration latency to productive infection [30]. Consequently, the rapid decay in the frequency of inducible infected cells we observed may reflect the fast *in vivo* turnover of cells in this transient state. To explore this hypothesis, we performed a post hoc analysis in a subset of 14 participants, assessing the dynamics of two-long-terminal repeat (2LTR) circles–a minor but representative form of uDNA [63] (S5 Fig). Overall, we observed a progressive decline in 2LTR circles over the first year of ART, with a median decrease from 145.6 copies per million cells at baseline to 21.2 at week 48 (median decay 84%), consistent with previously published data [25,33,64,65]. Notably, no significant decline was detected within the first 2 weeks of ART initiation. In fact, some participants showed a slight transient increase is 2LTR levels, a phenomenon previously described as a direct effect of integrase inhibitor treatment [33]. This kinetics profile diverges from the rapid decay in inducible infected cells observed in the same participants, suggesting that this swift clearance of the inducible reservoir is driven by mechanisms other than the turnover of cells harboring unintegrated viral forms.

One limitation of this study is that the VIP-SPOT assay may underestimate the frequency of inducible proviruses because not all of them are reactivated after a single round of stimulation, similar to the limitations observed with the QVOA [14,66]. However, the VIP-SPOT assay offers the benefits of being scalable and cost-effective. Another limitation concerns the composition of the study cohort, which consists only of men and harbors mostly subtype B infections, thus reflecting the current local epidemiology. This limitation prevents the analysis of potential differences in reservoir dynamics in women and other viral clades. Indeed, the study population is quite homogeneous, consisting entirely of ART-naive people starting a DTG-containing treatment regimen, thus resulting in very uniform trends in viral reservoir dynamics. These factors, along with the longitudinal and intensive sampling during the first weeks after treatment initiation, may account for differences between our results and those of other studies involving more heterogeneous cohorts and/or variable sampling [25,26]. Actually, the marked decline in the frequency of inducible proviruses may be facilitated by the use of integrase inhibitors in first-line antiretroviral regimens, as these agents can prevent the integration of extrachromosomal viral DNA forms that might otherwise contribute to the long-lived reservoir. This specific aspect could not be evaluated in our cohort, nor could the potential decline in the inducible reservoir beyond the first two weeks following ART initiation, as the median frequency (<1 HIV-1 antigen-producing cells per 10^6^ CD4^+^ T cells) was too close to the assay’s limit of detection to allow precise longitudinal assessment. Indeed, further reduction of the inducible reservoir may be mediated by CTLs capable of targeting cells expressing very low levels of viral p24–potentially below the detection threshold of the VIP-SPOT assay–or recognizing other viral antigens not evaluated in this study.

In conclusion, our investigation revealed rapid and specific clearance of cells harboring inducible proviruses within the first days after ART initiation. During this period, the interplay of opposing factors such as the reduction in viremia, the expansion of CD4^+^ T cells, and the restoration of host immune status collectively influenced the composition of the long-lived HIV-1 reservoir. Consequently, these findings support the idea that interventions focused on modulating establishment of viral latency may yield more significant benefits if implemented during this specific timeframe or immediately before ART initiation. Such approaches could further restrict the persistence of latently infected cells capable of triggering viral production upon ART interruption.

## Materials and methods

### Ethics statement

This study was approved by the Ethics Committee of the University Hospital Germans Trias i Pujol and all the participants provided their written informed consent.

### Study participants

The study population comprised participants from the DUALITY study [EudraCT number 2019-002733-10]. This randomized open-label clinical trial in ART-naïve people recently diagnosed with HIV-1 compared viral reservoir decay upon ART initiation with dolutegravir plus lamivudine (DTG + 3TC) (50 mg; 300 mg) or with DTG plus emtricitabine/tenofovir alafenamide (FTC/TAF) (50 mg; 200/25 mg). Participants were recruited at the Infectious Diseases Department of Hospital Germans Trias i Pujol (HUGTIP), Badalona, Spain, between October 2019 and October 2021. As results from this trial demonstrated no differential decay between the study arms in the HIV-1 reservoir parameters until week 48, all data were integrated for the present study [29].

Blood samples for storage of plasma and peripheral blood mononuclear cells (PBMCs) were collected at baseline (before ART initiation) and at weeks 1, 2, 4, and 12 and every 12 weeks thereafter until week 48.

### Standard and ultrasensitive viral load quantification

Plasma viral load (pVL) was monitored during the study period using the Alinity m HIV-1 assay (Abbot, USA). Once the pVL reached undetectable levels (<20 copies/mL), subsequent samples were retested to quantify residual viremia (down to 0.33 copies/mL) by ultracentrifugation of 9 mL of plasma followed by quantification using the Abbott Real-Time HIV-1 assay (Abbott Molecular Inc.).

### Proviral DNA analysis

CD4^+^ T cells were isolated from cryopreserved PBMCs by negative selection (CD4^+^ T Cell Isolation Kit, Miltenyi). Total HIV-1 DNA was measured using droplet digital PCR from an average of 7 × 10^5^ cells (ddPCR, Bio-Rad), with primer/probe sets targeting the 5’LTR and Gag regions, as previously described [67]. Likewise, intact proviruses, as well as proviruses with 3’ or 5’ deletions, were measured following the intact proviral DNA assay (IPDA) [22]. To obtain information on intactness from samples where the original IPDA primer/probe sets failed to amplify (14 out of 40), we used the abovementioned 5’LTR region or an alternative Env primer/probe set for samples failing the packaging signal (Ψ) or the Env Intact amplification, respectively [68]. Despite not retrieving information regarding the proportion of hypermutated proviruses, this strategy enabled us to estimate intactness data from 10 participants. The cellular *RPP30* gene was quantified to normalize the results (expressed as number of HIV-1 DNA copies per 10^6^ CD4^+^ T cells) and to correct for DNA shearing in the IPDA assay.

### Spontaneous viral transcription

Total RNA was extracted from 1-5 × 10^6^ isolated CD4^+^ T cells using the RNeasy Mini Kit (Qiagen). On average, 400 ng (range 150–1000 ng) was used to evaluate HIV-1 expression based on ddPCR with the One-Step RT-ddPCR Advanced Kit for probes (Bio-Rad). Initial 5’ elongation events were quantified by targeting 5’LTR or Gag, and the cellular housekeeping gene TATA-binding protein (*TBP*) was measured in parallel to normalize for cell input. All ddPCR determinations were performed with the QX100 Droplet Digital PCR System (Bio-Rad) and the QuantaSoft v1.6.6 software.

### Quantification of inducible proviruses

The frequency of cells retaining the capacity to revert from latency and produce viral proteins was quantified from an average of 3.3 × 10^6^ cells using the VIP-SPOT assay as described elsewhere [30]. Briefly, freshly isolated CD4^+^ T cells were cultured at 300,000 cells per well in ELISpot plates pre-coated with a capture anti-p24 antibody (Clone 39/5.4A, Zeptometrix) and activating anti-CD3 and anti-CD28 antibodies (clones OKT3 and CD28.2, eBioscience). Detection of viral p24 production 3 days later using a biotin-conjugated monoclonal antibody (clone 8G9, Novus Biologicals) enabled the quantification of HIV-antigen producer cells in each well. Images of the VIP-SPOT wells were captured with the ELISpot reader unit (Cellular Technology Limited), and spots counts were supervised manually.

### Mathematical modelling

The decay dynamics of proviral HIV-1 DNA, cell-associated HIV-1 RNA, and the inducible reservoir after ART initiation were analyzed using nonlinear mixed effects models. For each outcome, a 2-phase biexponential decay model was fitted, assuming a first rapid decay phase where productively infected cells are still present, and a second slow decay where productively infected cells were cleared. These models were run using the ‘nlme’ library in R v4.3.3. We used parametrization with 4 coefficients: the decay rates and the initial amplitudes for each of the 2 exponential components and, for each of those, both fixed and random effects were entered into the models. Goodness-of-fit was analyzed graphically based on the distribution of the residuals. Undetectable values were imputed to their limit of quantification. Predicted values obtained for each participant and marginal models (population-average) were plotted (S2–S4 Figs).

## Supporting information

S1 Fig Correlations between baseline values and data at week 48 are shown for total HIV-1 DNA (A), intact HIV-1 DNA (B), change in the percentage of intact provirus at week 48 relative to baseline and the levels of total proviral DNA (C) or intact provirus (D), cell-associated HIV-1 RNA (E), all expressed as copies per million CD4+ T cells/mL, and inducibility, expressed as percentage from infected cells (F). (TIFF)

S2 Fig Comparison of population-level (blue line) and individual-specific (orange line) estimated decay dynamics of pVL using non-linear mixed-effect models. Individual measures are also shown (dots).
(TIFF)

S3 Fig Comparison of population-level (blue line) and individual-specific (orange line) estimated decay dynamics of proviral HIV-1 DNA using non-linear mixed-effect models. Individual measures also shown (dots).
(TIFF)

S4 Fig Comparison of population-level (blue line) and individual-specific (orange line) estimated decay dynamics of the inducible reservoir using non-linear mixed-effect models. Individual measures also shown (dots).
(TIFF)

S5 Fig Quantification of 2LTR circles was performed using ddPCR in isolated CD4 + T cells from 14 participants. A. Individual values and median are shown for each time point. Asterisks indicate significant differences in 2LTR levels compared to baseline (Wilcoxon matched-pairs signed rank two-tailed test: *** p-value<0.001; ** p-value<0.01; * p-value<0.05). B. Longitudinal measures for individual participants are displayed in light red. Open symbols represent undetectable results, for which the value reported corresponds to half of the limit of detection.
(TIFF)

S1 Data Values File Tables including supporting data values for each main and supplementary figure are enclosed. (XLSX)
